# IL-2/IL-2 Receptor Pathway Plays a Crucial Role in the Growth and Malignant Transformation of HTLV-1-Infected T Cells to Develop Adult T-Cell Leukemia

**DOI:** 10.3389/fmicb.2020.00356

**Published:** 2020-03-06

**Authors:** Michiyuki Maeda, Junko Tanabe-Shibuya, Paola Miyazato, Hiroshi Masutani, Jun-ichirou Yasunaga, Kazumasa Usami, Akira Shimizu, Masao Matsuoka

**Affiliations:** ^1^Laboratory of Virus Control, Institute for Frontier Life and Medical Sciences, Kyoto University, Kyoto, Japan; ^2^Center for AIDS Research, School of Medicine, Kumamoto University, Kumamoto, Japan; ^3^Laboratory of Infection and Prevention, Institute for Frontier Life and Medical Sciences, Kyoto University, Kyoto, Japan; ^4^Department of Clinical Laboratory Sciences, Tenri Health Care University, Tenri, Japan; ^5^Institute for Advancement for Clinical and Translational Science, Kyoto University Hospital, Kyoto University, Kyoto, Japan; ^6^Taigenkai Hospital, Ichinomiya, Japan; ^7^Department of Hematology, School of Medicine, Kumamoto University, Kumamoto, Japan

**Keywords:** adult T-cell leukemia, human T-cell leukemia virus type 1, IL-2/IL-15/IL-2R, Tax, HBZ, p53, growth progression

## Abstract

T cells infected with human T-cell leukemia virus type 1 (HTLV-1) transform into malignant/leukemic cells and develop adult T-cell leukemia (ATL) after a long latency period. The *tax* (transactivator from the X-gene region) and *HBZ* (HTLV-1 bZIP factor) genes of HTLV-1 play crucial roles in the development of ATL. The process and mechanism by which HTLV-1-infected T cells acquire malignancy and develop ATL remain to be elucidated. Constitutive expression of interleukin-2 (IL-2) receptor α-chain (IL-2Rα/CD25), induced by the *tax* and *HBZ* genes of HTLV-1, on ATL cells implicates the involvement of IL-2/IL-2R pathway in the growth and development of ATL cells *in vivo*. However, the leukemic cells in the majority of ATL patients appeared unresponsive to IL-2, raising controversies on the role of this pathway for the growth of ATL cells *in vivo*. Here, we report the establishment of 32 IL-2-dependent T-cell lines infected with HTLV-1 from 26 ATL patients, including eight leukemic cell lines derived from five ATL patients, while no T-cell lines were established without IL-2. We have shown that the IL-2-dependent ATL cell lines evolved into IL-2-independent/-unresponsive growth phase, resembling ATL cells *in vivo*. Moreover, the IL-2-dependent non-leukemic T-cell lines infected with HTLV-1 acquired IL-2-independency and turned into tumor-producing cancer cells as with the ATL cell lines. HTLV-1-infected T cells *in vivo* could survive and proliferate depending on IL-2 that was produced *in vivo* by the HTLV-1-infected T cells of ATL patients and patients with HTLV-1-associated diseases and, acts as a physiological molecule to regulate T-cell growth. These results suggest that ATL cells develop among the HTLV-1-infected T cells growing dependently on IL-2 and that most of the circulating ATL cells progressed to become less responsive to IL-2, acquiring the ability to proliferate without IL-2.

## Introduction

A unique T-cell leukemia endemic to the southwestern district of Japan was described in the early 1970s and was reported as ATL in 1977 (Yodoi et al., 1974; Uchiyama et al., 1977). In 1980, the first human retrovirus HTLV-1 was isolated from a patient of Caribbean origin with an aggressive type of mycosis fungoides (later identified as ATL), and another human retrovirus, HTLV-2, was isolated from a patient with hairy cell leukemia in 1981 (Poiesz et al., 1980; Gallo, 2005). Independently, a retrovirus was also detected in a T-cell line derived from a Japanese patient with ATL. The retrovirus infection was detected in all patients clinically suspected or diagnosed with ATL and some healthy individuals by using this cell line in 1981 (Hinuma et al., 1981; Miyoshi et al., 1981a; Yoshida et al., 1982).

HTLV-1 isolated from geographically separated districts were shown to be identical at the sequence level.

Monoclonal proliferation of an HTLV-1-infected T-cell clone, mostly CD4(+) T cells with integrated HTLV-1 provirus in each ATL patient’s leukemic cells, provided evidence for HTLV-1 as the causative agent of ATL (Hinuma et al., 1981; Yoshida et al., 1984). In ATL patients and healthy HTLV-1 carriers, polyclonal T cells were infected with HTLV-1 (Etoh et al., 1997; Gillet et al., 2011).

Adult T-cell leukemia is estimated to have developed in 2–7% of HTLV-1-infected people after 40–60 years and the lifetime risk of developing ATL is estimated to be 6–7% for men and 2–3% for women infected with HTLV-1 in Japan (Iwanaga et al., 2012).

The *tax* and *HBZ* genes of HTLV-1 have been shown to induce leukemia/cancer and inflammatory diseases in transgenic mice, as well as cause the transformation/immortalization of T cells *in vitro* (Grassmann et al., 1989; Grossman et al., 1995; Hasegawa et al., 2006; Satou et al., 2006; Boxus et al., 2008; Satou et al., 2011). How a T-cell clone within the polyclonal T cells infected with HTLV-1 acquires malignancy and develops ATL after a long latency period remains to be elucidated. Furthermore, the molecular mechanism of ATL cell proliferation *in vivo* remains to be elucidated too.

IL-2-receptor α-chain (IL-2Rα)/CD25 expressed constitutively on the cell surface (Hattori et al., 1981) suggested the involvement of IL-2/IL-2R in the growth of ATL cells. Indeed, we and other researchers established a number of T-cell lines infected with HTLV-1 using IL-2 from ATL patients. Most of them were non-leukemic T-cell lines, but there was still a significant number of leukemic cell lines as well (Gazdar et al., 1980; Miyoshi et al., 1980, 1981a, 1981b; Hoshino et al., 1983; Sugamura et al., 1984; Maeda et al., 1985, 1987; Arima et al., 1986; Katoh et al., 1986; Yamada et al., 1998). Thus, we proposed that ATL cell lines were derived from IL-2-dependent/responsive cells *in vivo* and progressed to be IL-2 independent. Since the majority of ATL cells were reported to be unresponsive to IL-2, the IL-2-dependent proliferation of ATL cells has been questioned. Some of these IL-2-dependent leukemic and non-leukemic cell lines, however, became to proliferate without IL-2 and were unresponsive to IL-2, resembling ATL cells *in vivo*.

Based on the statistical analysis of the mode of age-specific occurrence of ATL, a multistep growth progression hypothesis was proposed for the development of ATL cells from normal T cells infected with HTLV-1 (Okamoto et al., 1989). The integrated gene analysis of ATL cells isolated from a number of ATL patients indicates that ATL cells arise after the accumulation of multiple genomic alterations in HTLV-1-infected T cells, supporting the multistep development of ATL (Kataoka et al., 2015).

In the present report, we provide supporting evidence for the involvement and importance of IL-2/IL-2R in the growth and the malignant transformation of HTLV-1-infected T cells into ATL cells *in vivo* to develop ATL.

## Results

### Establishment of IL-2-Dependent Leukemic and Non-leukemic T-Cell Lines From ATL Patients and Patients With HTLV-1-Associated Diseases

Thirty-two IL-2-dependent T-cell lines infected with HTLV-1 were established from the PBMCs of 26 ATL patients in the presence of IL-2.

**FIGURE 1:**
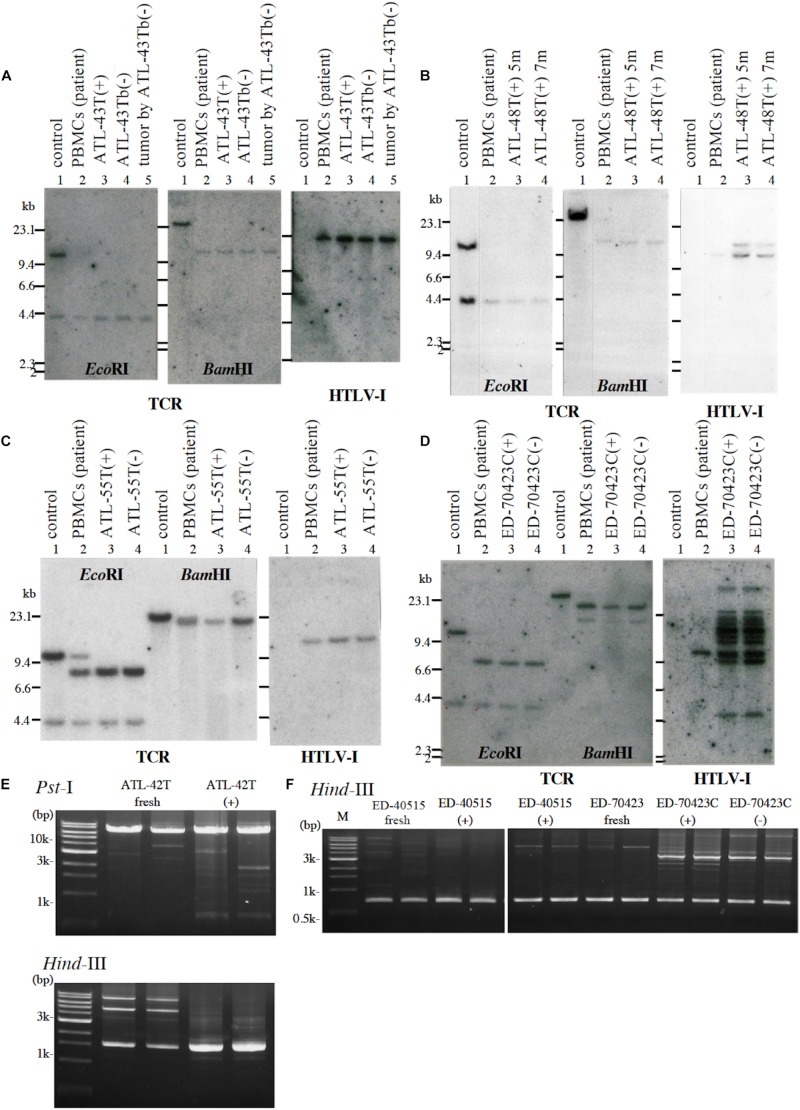
Profiles of *T-cell receptor β* chain (*TCRβ*) gene rearrangement and/or provirus integration sites in five leukemic cell lines examined by Southern blot or reverse PCR analysis. **(A)** *TCRβ* chain gene rearrangement and provirus integration sites are shown for ATL-43T cells. DNA isolated from a B-cell line (lane 1), not-rearranged control, PBMCs of an ATL patient (lane 2), IL-2-dependent (lane 3), IL-2-independent (lane 4) T-cell line derived from the ATL patient, and a tumor produced by IL-2-independent T-cell line (lane 5). **(B)** *TCRβ* chain gene rearrangement and provirus integration sites are shown for ATL-48T cells. DNA was isolated from a B-cell line (lane 1), not-rearranged control, and PBMCs of an ATL patient (lane 2), the IL-2-dependent T-cell lines cultured for 5 (lane 3) and 7 months (lane 4). The same analysis was performed for ATL-55T **(C)** and ED-70423C **(D)**. DNA was digested with *Eco*RI and *Bam*HI as shown. Profiles of the provirus integration site were examined by inverse PCR analysis for ATL-42T **(E)** and ED-40515 and ED-70423 cells **(F)**. DNA was digested with *Pst*I and/or *Hin*dIII **(E)**. In **F**, provirus integration sites were examined in the established leukemic cell lines derived from ED-40515 and ED-70423C cells too.

Among them, eight IL-2-dependent leukemic cell lines, which have the same TCRβ chain gene rearrangement and HTLV-1 provirus integration site as the leukemic cells in the patient, were established from five ATL patients (Figures 1A–E). Four of these leukemic cell lines were established from a patient with chronic ATL who had been initially diagnosed as suffering from erythroderma (ED) and was infected with HTLV-1 during the clinical course over 3 years.

Following the establishment of a leukemic ED-40515(+) cell line (Maeda et al., 1985), two more leukemic cell lines, ED-40810S(+) and ED-41214C(+), were established from the same patient’s PBMCs isolated 3 and 7 months later, respectively (Maeda et al., 1985, 1987). One additional leukemic ED-70423C(+) cell line was established from the PBMCs of the patient (white blood cells: WBC; 197,800/μl, atypical lymphocytes; 75%) in acute exacerbation, isolated after 3 years of the onset of ATL (Figures 1D,F). The leukemic cells of this patient were sustained clonally identical over the 3 years of the patient’s clinical course (Figure 1F). These four IL-2-dependent leukemic cell lines acquired IL-2-independency between 6 and 22 months after the initiation of each cell culture, and were designated ED-40515(−), ED-40810S(−), ED-41214C(−), and ED-70423C(−). These leukemic T-cell lines derived from ATL cells of the patient had the same TCRβ chain gene rearrangement as the leukemic cells (Supplementary Figure S1 and Figure 1D).

**FIGURE 2:**
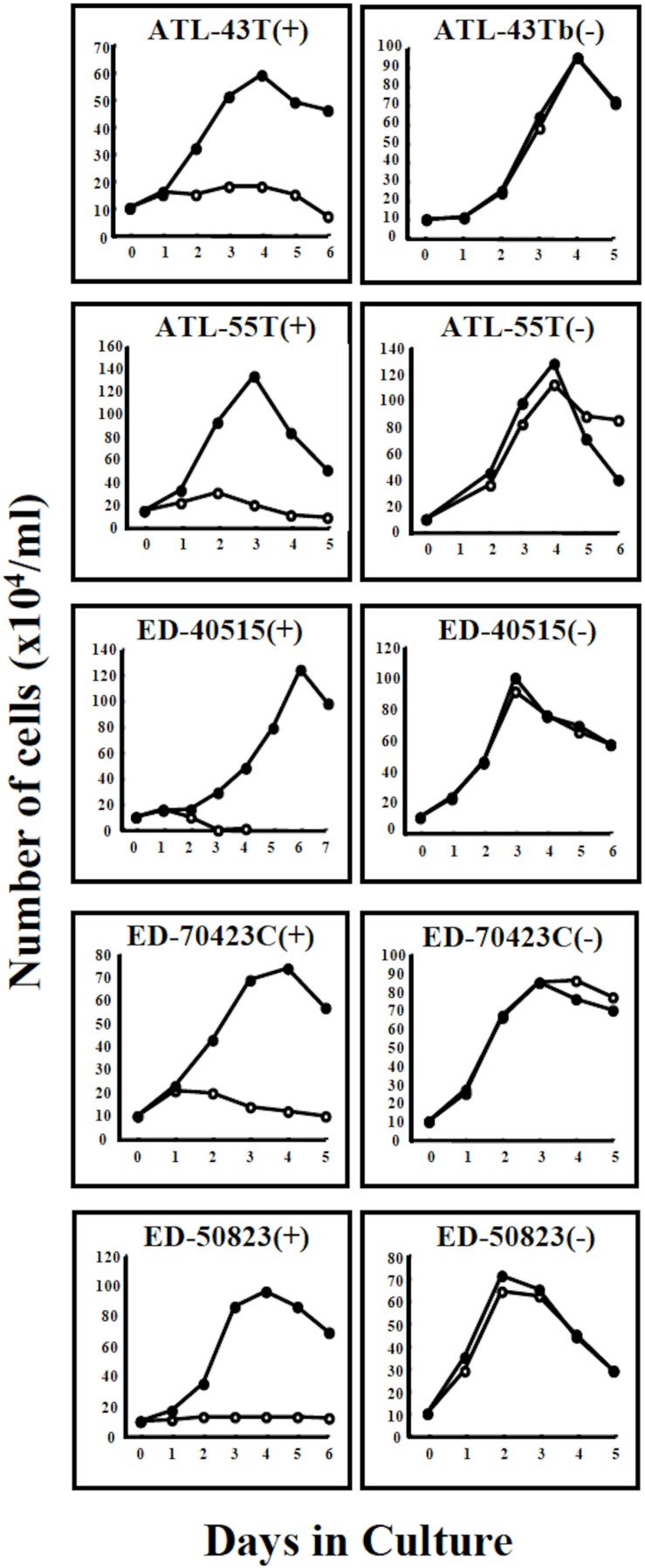
Growth curves of 4 ATL cell lines and a non-leukemic cell line in IL-2-dependent and IL-2-independent growth phase. Four IL-2-dependent leukemic cell lines, ATL-43T(+), ATL-55T(+), ED-40515(+), and ED-70423C(+) are shown to have acquired IL-2-independency. A non-leukemic ED-50823(+) cell line also acquired IL-2-independency. Cells were cultured with (🌑-🌑) or without (○-○) IL-2.

A non-leukemic T-cell line, ED-50823(+), with the TCRβ chain gene rearrangement and HTLV-1 provirus integration site different from the leukemic cells, was also established from this same patient’s PBMCs in remission (WBC; 9,900/μl, 4%), isolated 15 months after the diagnosis of ATL (Figure 2). The leukemic cells of this patient proliferated in the presence of IL-2 for a month, but subsequently the monoclonal non-leukemic T cells overtook the culture. The leukemic cells were not detected after 4 months in culture. The IL-2-independent ED-50823(−) line was also isolated (Figure 2).

T-cell lines in IL-2-dependent and IL-2-independent growth phases were designated with the suffixes (+) and (−) following the cell line name.

**FIGURE 3:**
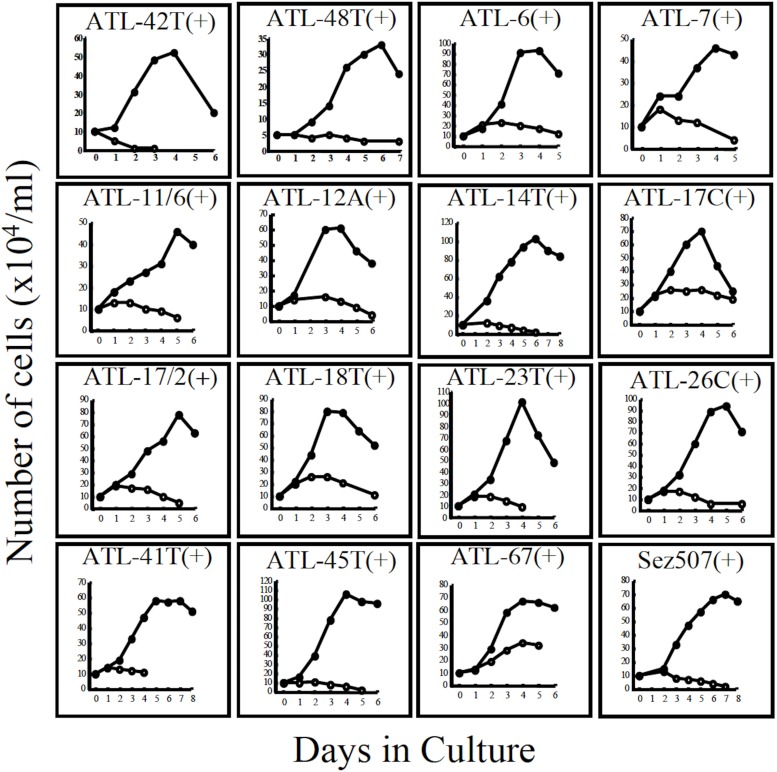
Growth curves of 16 IL-2-dependent T-cell lines infected with HTLV-1 derived from 15 ATL patients and a non-ATL patient infected with HTLV-1. ATL-42T(+) and ATL-48T(+) are leukemic cell lines. Others are non-leukemic T-cell lines. Sez507(+) is a HTLV-1(+) T-cell line derived from a patient with Sezary syndrome-like skin disease. Cells were cultured with (🌑-🌑) or without (○-○) IL-2.

In addition to these ATL cell lines, here we report the establishment of another 4 leukemic cell lines from four ATL patients. ATL-43T(+) was established from an acute ATL patient with CD4(−)CD8(−)CD25(+) and TCRαβ(+) leukemic cells of gastrointestinal origin (WBC; 17,100/μl, 31%) (Figures 1A, 2). ATL-55T(+) was established from an acute ATL patient with CD4(+) leukemic cells (WBC; 83,400/μl, 87%) (Figures 1C, 2). ATL-48T(+) and ATL-42T(+) were established from two chronic ATL patients with CD4(+) leukemic cells (WBC; 5,100/μl, 30% and 10,800/μl, 2%) (Figures 1B,E, 3). IL-2-independent ATL-43Tb(−) and ATL-55T(−) cell lines were isolated (Figures 1A,C, 2), but no IL-2-independent cell lines have been isolated from ATL-42T(+) and ATL-48T(+) cell lines. Characteristics of these newly established leukemic cell lines are summarized in Table 1.

**TABLE 1:** Leukemic cell lines established from the peripheral blood mononuclear cells of ATL patients.

**Leukemic cell line**	**Type of leukemia (leukemic cell phenotype)**	**Number of WBC (atypical cell%) in the patient’s PBMC**	**Progression to IL-2-independent growth phase**	**Progression to tumor producer cell**
ED-70423C(+)	Chronic (CD4 +)	197,800/μl (75)	+	+
ATL-42T(+)	Chronic (CD4 +)	10,800 (2)	–	–
ATL-43T(+)	Acute (CD4-CD8-)	17,100 (31)	+	+
ATL-48T(+)	Chronic (CD4 +)	5,100 (30)	–	–
ATL-55T(+)	Acute (CD4 +)	83,400 (87)	+	Not tested

*Five leukemic cell lines were established from the peripheral blood mononuclear cells (PBMC) of five ATL patients in the presence of IL-2. WBC and the percentage of atypical cells in the patient’s blood sample used for cell culture are shown. Progression of IL-2-dependent phase to IL-2-independent and tumor producer phase of these cell lines is shown.*

Interestingly, 24 cell lines out of the 32 IL-2-dependent T-cell lines (24/32:75%) established from 26 ATL patients were derived from non-leukemic T cells infected with HTLV-1 in the patient’s PBMCs. A list of these IL-2-dependent leukemic and non-leukemic T-cell lines is shown in Table 2.

**TABLE 2:** HTLV-1-infected T-cell lines established from ATL patients.

**Cell line name**	**Patient No.**	**Conversion to IL-2-independent**	**Leukemic or non-1 eukemic**	**Phenotype CD4/CD8**
ED-40515	#1	+	Leukemic	CD4+
ED-40810S	#1	+	Leukemic	CD4+
ED-41214T	#1	+	Leukemic	CD4+
ED-70423C	#1	+	Leukemic	CD4+
ED-50823	#1	+	Non-Leukemic	CD4+
ATL-42T	#2	−	Leukemic	CD4+
ATL-43T	#3	+	Leukemic	CD4−/CD8−
ATL-48T	#4	−	Leukemic	CD4+
ATL-55T	#5	+	Leukemic	CD4+
ATL-2	#6	+	Non-Leukemic	CD4+
ATL-6	#7	−	Non-Leukemic	CD4+
ATL-7	#8	−	Non-Leukemic	CD4+
ATL-11-6	#9	−	Non-Leukemic	CD4+
ATL12A	#10	−	Non-Leukemic	CD4+
ATL-13	#11	−	Non-Leukemic	CD4+
ATL-14T	#12	−	Non-Leukemic	CD4+
ATL-16T	#13	+	Non-Leukemic	CD4+
ATL-17C	#14	−	Non-Leukemic	CD4+
ATL-17-2	#14	−	Non-Leukemic	CD4+
ATL-18T	#15	−	Non-Leukemic	CD4+
ATL-20	#16	−	Non-Leukemic	CD4+
ATL-21C	#17	+	Non-Leukemic	CD4−/CD8−
ATL-23T	#18	−	Non-Leukemic	CD4+
ATL-26C	#19	−	Non-Leukemic	CD4−/CD8−
ATL-35T	#20	+	Non-Leukemic	CD4+
ATL-40T	#21	−	Non-Leukemic	CD4−/CD8−
ATL-41T	#22	−	Non-Leukemic	CD4+
ATL-45T	#23	−	Non-Leukemic	CD4+
ATL-46T	#24	−	Non-Leukemic	CD4+
ATL-67T	#25	−	Non-Leukemic	CD4+
ATL-72b	#26	+	Non-Leukemic	CD4+
ATL-72/2	#26	+	Non-Leukemic	CD4+

*Thirty-two HTLV-1(+) T-cell lines were established in the presence of IL-2 from the peripheral blood mononuclear cells of 26 ATL patients. The origin (leukemic or non-leukemic), conversion to IL-2-independency, and the phenotype (CD4/CD8) are shown.*

In addition, six IL-2-dependent T-cell lines were established from the PBMCs of a patients with Sézary syndrome-like skin disease infected with HTLV-1, a patient with HAM/TSP, and two healthy carriers of HTLV-1 in the presence of IL-2, indicating that HTLV-1-infected T cells in patients with HTLV-1-associated diseases and healthy carriers of HTLV-1 were also able to proliferate continuously in the presence of IL-2. A list of these six HTLV-1-infected T-cell lines is shown in Supplementary Table S1.

The growth curves of two leukemic and 13 non-leukemic T-cell lines established from ATL patients and, one T-cell line, Sez507, derived from patient with Sezary-syndrome like skin disease infected with HTLV-1, all of them IL-2-dependent, are shown in Figure 3.

Without IL-2, no HTLV-1-infected T-cell lines could be established from the PBMCs of ATL patients, patients with HTLV-1-associated diseases and healthy carriers of HTLV-1.

T cells infected with HTLV-1 were confirmed to express IL-2Rα and proliferate continuously *in vitro* in the presence of IL-2.

**FIGURE 4:**
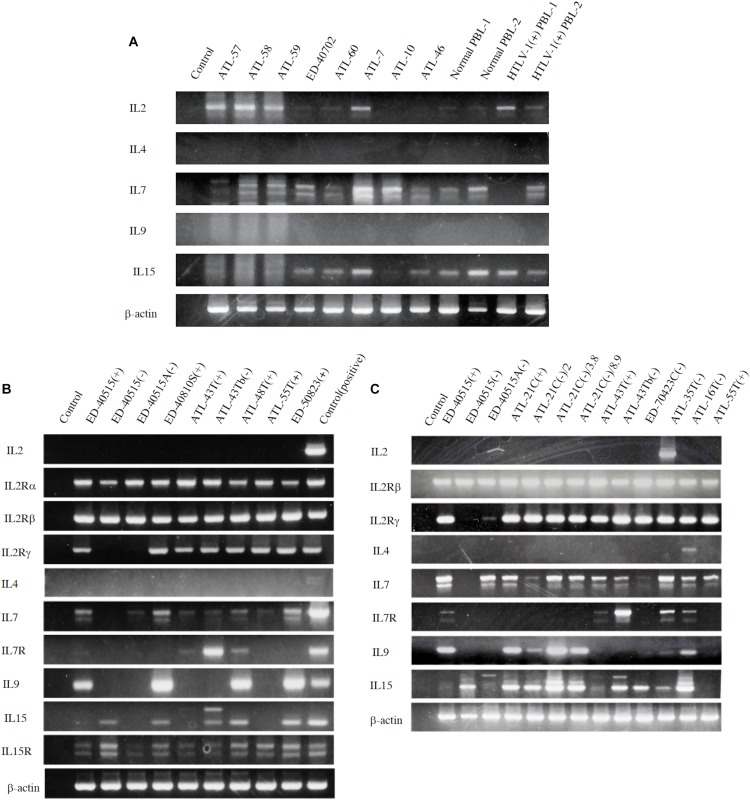
Expression of T-cell cytokine/cytokine-receptor genes in the PBMCs of ATL patients and HTLV-1 carriers *in vivo*, and the HTLV-1-infected T-cell lines. T-cell cytokine/cytokine-receptor gene expression was examined by RT-PCR in the PBMCs isolated from eight ATL patients, two HTLV-1 healthy carriers, and two healthy people **(A)**, and in the ATL cell lines and HTLV-1-infected T-cell lines established from ATL patients **(B,C)**. The names of examined cells and cell lines are shown on each column. The names of cytokines and their receptors tested are shown on the left side of each figure. The results of two independent experiments are shown for ATL-derived T-cell lines **(B,C)**. *β-actin* gene was used as the positive control of gene expression. The reproducibility of the results was confirmed by the experiment more than twice.

**FIGURE 5:**
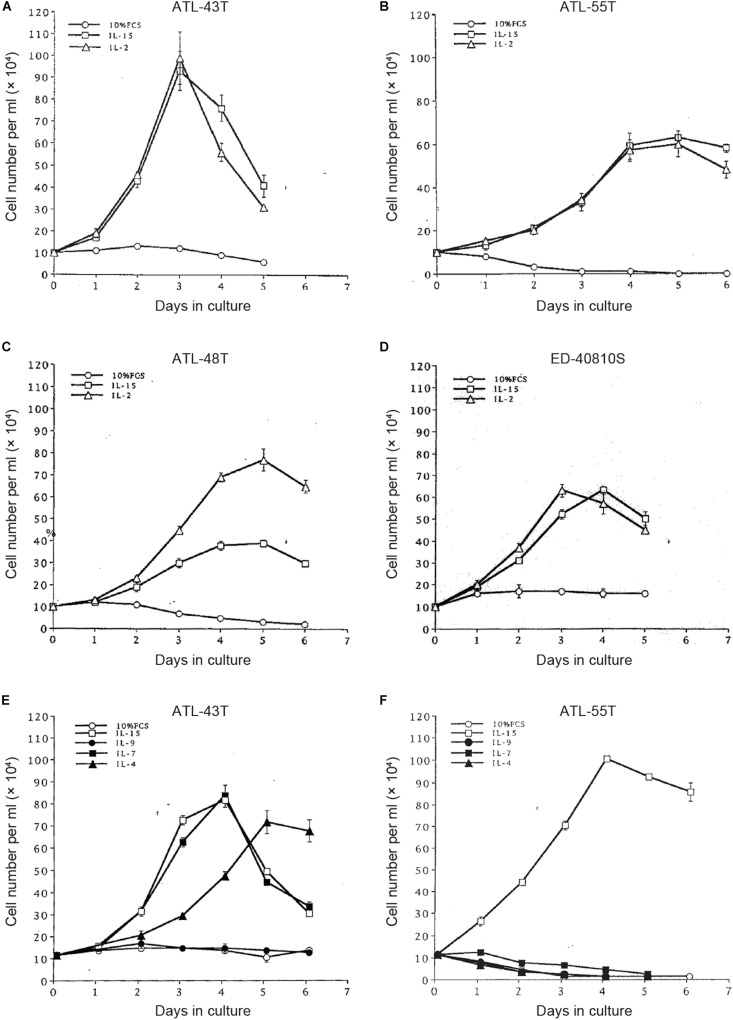
Growth stimulation of IL-2-dependent ATL cell lines by IL-15, -9,-7 and IL-4. Four IL-2-dependent ATL cell lines were depleted IL-2 and were cultured with IL-2 or IL-15 **(A–D)**. Two IL-2-dependent ATL cell lines were cultured with IL-15, -9, -7 or IL-4 **(E,F)**.

However, to proliferate and survive *in vivo*, HTLV-1-infected T cells should be provided IL-2. We examined whether the PBMCs of ATL patients and healthy carriers of HTLV-1 could be the source of IL-2 *in vivo*. The PBMCs of four ATL patients out of eight patients and, a healthy HTLV-1 carrier, were shown to express IL-2 mRNA, suggesting that HTLV-1-infected T cells produce IL-2 *in vivo* (Figure 4A). Messenger RNA of IL-7 and IL-15 were also detected, suggesting the involvement of IL-15 and IL-7 in the growth of HTLV-1-infected T cells (Figures 4A, 5).

### Growth Evolution of HTLV-1-Infected T-Cell Line Cells Into Tumor-Producing Cancer Cells

In the pathogenesis of ATL, normal T cells infected with HTLV-1 undergo malignant transformation into leukemic cells. To demonstrate the involvement of IL-2/IL-2R in the growth of HTLV-1-infected T cells, we established IL-2-dependent T-cell lines from ATL patients.

By using these IL-2-dependent T-cell lines infected with HTLV-1, we examined whether HTLV-1-infected T cells growing dependently on IL-2, progress to grow in IL-2-independent manner and whether they evolve into tumor-producing malignant cells.

Four IL-2-dependent leukemic cell lines and a non-leukemic T-cell line were shown to progress from an IL-2-dependent to IL-2-independent growth phase (Figure 2).

In total, six out of 8 IL-2-dependent leukemic cell lines (6/8:75%), and seven out of 24 IL-2-dependent non-leukemic T-cell lines (7/24:29%) established from ATL patients acquired IL-2-independency, resembling ATL cells *in vivo* (Table 2).

To examine the possible involvement of a cytokine(s) in the transition from IL-2-dependent to IL-2-independent growth phase, these T-cell lines were surveyed for the presence of mRNA of *T-cell cytokine/cytokine receptor* genes. Messenger RNA of *IL-7* and *IL-15/IL-15R* genes, but not *IL-2* gene, was expressed in the majority of HTLV-1-infected T-cell lines (Figures 4B,C), although no significant growth stimulating activity for the IL-2-dependent T-cell lines was detected in the culture supernatant of the IL-2-independent T cells. However, four IL-2-dependent leukemic T-cell lines, ATL-43T(+), ATL-55T(+), ATL-48T(+), and ED-40810S(+), could proliferate in the presence of IL-15 instead of IL-2, and ATL-43T(+) could proliferate in the presence of IL-7 and IL-4 (Figure 5).

Then, to examine whether or not these HTLV-1-infected T-cell lines could acquire malignancy, viable cells were inoculated into immunodeficient nude and/or severe combined immunodeficiency (SCID) mice to check for tumor formation as an evidence of malignancy. The results are summarized in Table 3.

**TABLE 3:** Tumorigenicity of non-leukemic and leukemic T-cell lines derived from ATL patients.

**Cell lines**	**No. of injected cells**	**SCID mice**		**Nude mice**	
		**No. of tumors/No. of injected sites**	**Tumor weight (g, mean ± SD)**	**No. of tumors/No. of injected sites**	**Tumor weight (g, mean ± SD)**
ATL-21C(+)	1 × 10^7^	0/8	–	0/8	–
ATL-21C(−)/2	2 × 10^7^	3/4*	0.4 (*n* = 2)	n.t	–
	1 × 10^7^	0/14	–	0/14	–
ATL-21C(−)/3.8	1 × 10^7^	8/8	1.9 ± 1.5 (*n* = 4)	8/8	0.5 ± 0.4 (*n* = 6)
ATL-21C(−)/8.9	1 × 10^7^	8/8	1.0 ± 0.5 (*n* = 6)	6/8	0.2 ± 0.1 (*n* = 4)
ATL-16T(+)	1 × 10^7^	0/7	–	0/7	–
ATL-16T(−)	1 × 10^7^	7/15	1.9 ± 1.6 (*n* = 4)	0/11	–
ATL-35T(−)	1 × 10^7^	4/8	0.4 ± 0.3 (*n* = 4)	0/4	–
ED-40515(+)	2 × 10^7^	0/16	–	n.t.	–
	1 × 10^7^	0/8	–	0/8	–
ED-40515(−)	2 × 10^7^	3/3*	n.t.	23/24	2.1 ± 1.8 (*n* = 5)
	1 × 10^7^	6/6	3.0 ± 0.6 (*n* = 6)	6/6	3.6 ± 2.4 (*n* = 6)
	2 × 10^6^	2/2	9.8 (*n* = 2)	2/2	2.5 (*n* = 2)
	1 × 10^6^	2/2	0.8 (*n* = 2)	n.t.	
ED-70423C(+)	1 × 10^7^	0/8	–	0/8	–
ED-70423C(−)	2 × 10^7^	2/8*	n.t.	n.t.	–
	1 × 10^7^	0/8	–	0/8	–
ATL-43T(+)	1 × 10^7^	0/14	–	0/14	–
ATL-43Tb(−)	2 × 10^7^	4/4	1.3 ± 0.8 (*n* = 3)	n.t.	–
	1 × 10^7^	3/4	2.0 ± 1.0 (*n* = 3)	4/4	1.1 ± 1.5 (*n* = 4)
ATL-48T(+)	1 × 10^7^	0/8	–	n.t.	–
ATL-55T(+)	1 × 10^7^	0/8	–	0/8	–

Three non-leukemic (ATL-21C, ATL-16T, and ATL-35T) and five leukemic (ED-40515, ED-70423C, ATL-43T, ATL-48T, and ATL-55T) T-cell line cells in IL-2-dependent and IL-2-independent growth phase were injected subcutaneously into 4–6-week-old male SCID mice and/or BALB/c nude mice. Tumor mass formation was examined at 1–1.5 months after cell injection. Tumor weight more than 0.1 g is counted tumor positive and is shown as mean ± SD. IL-2-dependent and IL-2-independent cell lines are designated by (+) and (−) suffixed to each cell line name, respectively. *Intraperitoneal inoculation. n.t.: not tested.

**FIGURE 6:**
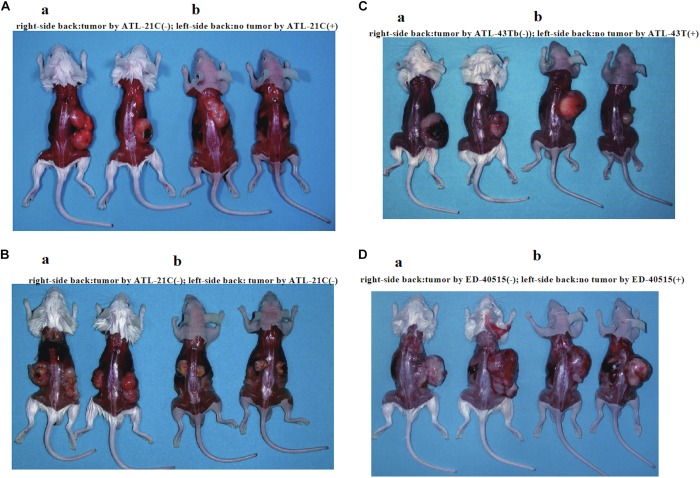
Tumors produced in immunodeficient mice by a non-leukemic T-cell line (ATL-21C) and two leukemic cell lines [ED-40515(−) and ATL-43Tb(−)] derived from ATL patients. Viable cells (1 × 10^7^) were inoculated subcutaneously on the back of both sides (**a:** SCID mouse, **b:** Nude mouse) and tumor mass formation was examined after 1–1.5 months. **(A)** Tumors produced by ATL-21C(−)/3.8 T-cells (right side). No tumor production by ATL-21C(+) T-cells (left side). **(B)** Tumors produced by ATL-21C(−)/8.9 T-cells on both sides. **(C)** Tumors produced by ATL-43Tb(−) cells (right side) and no tumor production by ATL-43T(+) cells (left side). **(D)** Tumors produced by ED-40515(−) (right side) and no tumor production by ED-40515(+) (left side). The reproducibility of the results was confirmed by performing the experiment more than twice.

**FIGURE 7:**
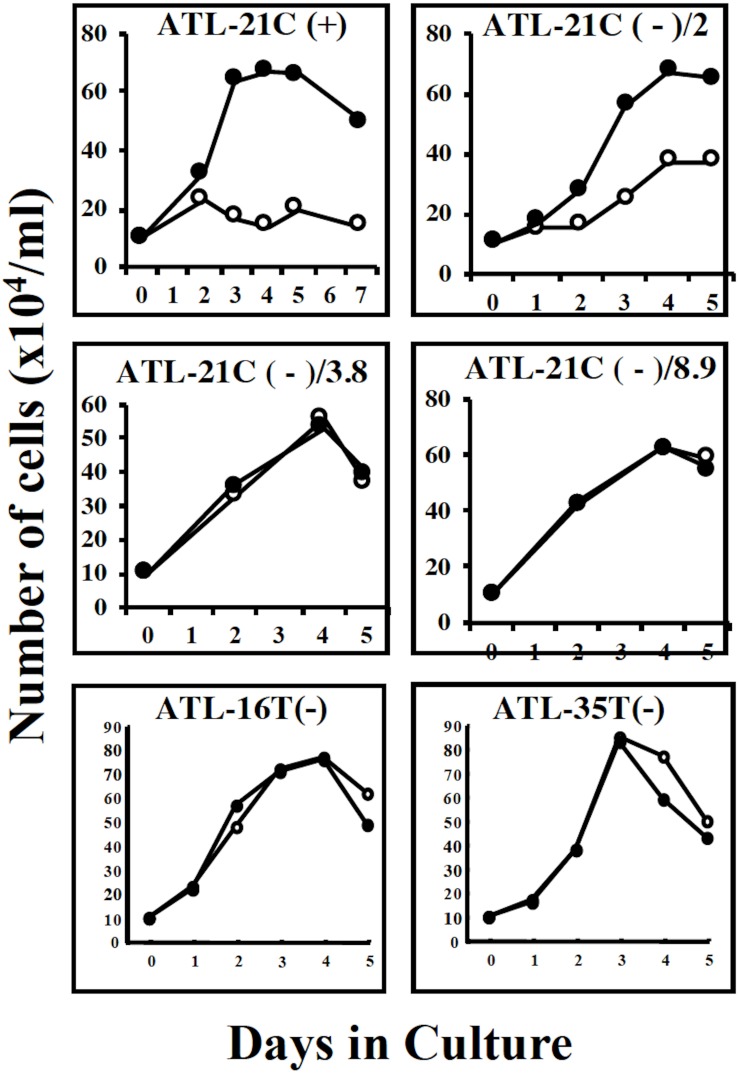
Growth curves of three tumor-producing non-leukemic T-cell lines in the IL-2-dependent/-independent growth phase. Cells were cultured with (🌑-🌑) or without (○-○) IL-2. ATL-21C(+)/ATL21C(−), ATL-16(−), and ATL-35T(−) were examined. ATL-21C(+), IL-2-dependent phase. ATL-21C(−)/2, IL-2-independent, but IL-2 responsive, cultured for 2 years. ATL-21C(−)/3.8, cultured for 3yr8mo. ATL-21C(−)/8.9, cultured for 8yr9mons.

**FIGURE 8:**
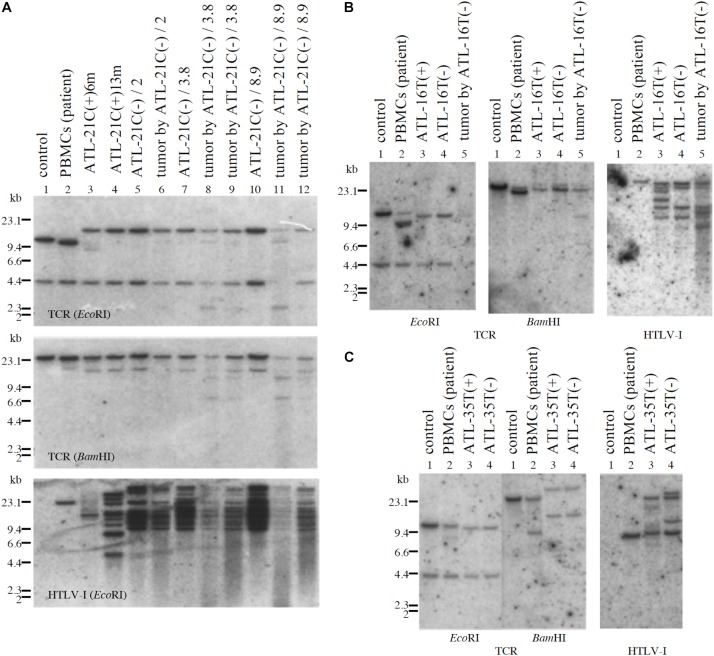
Profiles of *TCR*-β gene rearrangement and HTLV-1 provirus integration sites in three tumor-producing non-leukemic T-cell lines established from ATL patients. *TCR*-β chain gene rearrangement and provirus integration sites were examined by Southern blot analysis. *TCR*-β chain gene rearrangement and provirus integration sites are shown for ATL-21C **(A)**, ATL-16T **(B)**, and ATL-35T **(C)** cells. **(A)** Lane 1: B-cell line (control, non-rearranged cells), 2: the ATL patient’s primary PBMCs (leukemic cells), 3: ATL-21C(+) in early culture (6 months) derived from the patient, 4: ATL-21C(+) in advanced culture (13 months), 5: ATL-21C(−)/2, 6: tumor induced in SCID mouse by ATL-21C(−)/2, 7: ATL-21C(−)/3.8, 8: tumor induced in nude mouse by ATL-21C(−)/3.8, 9: tumor induced in SCID mouse by ATL-21C(−)/3.8, 10: ATL-21C(−)/8.9, 11: tumor induced in nude mouse by ATL-21C(−)/8.9, and 12: tumor induced in SCID mouse by ATL-21C(−)/8.9. **(B)** Lane 1: B-cell line, 2: the ATL patient’s primary PBMCs (leukemic cells), 3: ATL-16T(+) derived from the patient, 4: ATL-16T(−), and 5: tumor induced in SCID mouse by ATL-16T(−). **(C)** Lane 1: B-cell line, 2: the ATL patient’s primary PBMCs (leukemic cells), 3: ATL-35T(+) derived from the patient, and 4: ATL-35T(−). DNA was digested with EcoRI and BamHI. The reproducibility of the results was confirmed by performing the experiment more than twice.

ATL-21C(−), ATL-16T(−), and ATL-35T(−) cell lines produced tumors in immunodeficient mice (Table 3 and Figures 6A,B). ATL-21C(+) is an IL-2-dependent non-leukemic T-cell line established from the PBMCs of an acute ATL patient with CD4(+)CD8(+) leukemic cells. ATL-21C cells cultured in the presence of IL-2 for 2 years were found to proliferate without IL-2, being slightly responsive to IL-2 (Figure 7). Thereafter, ATL-21C T-cell lines were maintained in culture without IL-2. ATL-21C T-cell lines that have been maintained in culture for a total of 2 years, 3 years 8 months, and 8 years 9 months, designated as ATL-21C(−)/2, ATL-21C(−)/3.8, and ATL-21C(−)/8.9, were shown to grow without IL-2 (Figure 7). These ATL-21C T-cell lines showed to have made growth progression from IL-2-dependent to IL-2-independent growth phase and further evolved into tumor-producing cancer cells in immunodeficient mice as the same clonal lines (Figure 8A and Table 3). ATL-21C cell lines express CD25 and HLA-DR antigens, hallmarks of HTLV-1-infected T-cells, but the expression of CD4/CD8 antigens was lost during long-term culture (Supplementary Figure S2). ATL-16T(+) and ATL-35T(+) were established from a chronic and an acute ATL patient with CD4(+) leukemic cells, respectively, and acquired IL-2-independency after 9 and 10 months in culture, respectively (Figures 7, 8B,C). ATL-16T(−) and ATL-35T(−) cell lines produced tumors in immunodeficient mice (Table 3 and Figure 8B).

Three leukemic T-cell lines, ED-40515(−), ED-70423C(−), and ATL-43Tb(−), produced tumor masses in immunodeficient mice (Table 3 and Figures 6C,D). However, both IL-2-dependent non-leukemic and leukemic T-cell lines produced no tumors in immunodeficient mice (Figures 6C,D).

We did not test the survival of these cells in mice by injecting human IL-2.

### Expression of Tax, HBZ, and p53 Genes in the Non-leukemic and Leukemic T-Cell Lines Derived From ATL Patients

To clarify whether or not the *tax*, *HBZ* and *p53* genes were involved in the establishment and the malignant transformation of the HTLV-1-infected T-cell lines, mRNA expression of these genes was examined on these T-cell lines.

**FIGURE 9:**
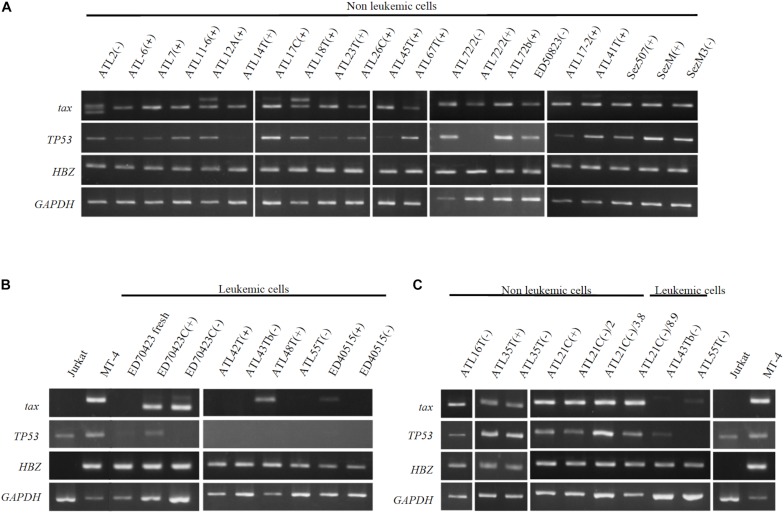
Profiles of *tax*, *p53*, and *HBZ* genes expressed in T-cell lines derived from ATL patients. Transcripts of *tax*, *p53*, and *HBZ* genes in the leukemic and non-leukemic T-cell lines derived from ATL patients and people infected with HTLV-1 were examined by RT-PCR. RNA was isolated from: **(A)** Non-leukemic T-cell lines. **(B)** Leukemic T-cell lines. **(C)** Non-leukemic tumor-producer ATL-21C(−), ATL-16T(−), and ATL-35T(−) T-cell lines. Jurkat and MT-1 cell lines were used as negative and positive controls for *tax* and *HBZ* gene’ expression, respectively. *GAPDH* gene was used as the positive control of gene expression. The reproducibility of the results was confirmed by performing the experiment more than twice.

The *tax* gene transcript of the size predicted was detected by RT-PCR in 20 IL-2-dependent and nine IL-2-independent non-leukemic T-cell lines infected with HTLV-1 established from 19 ATL patients, a patient with Sézary syndrome-like skin disease infected with HTLV-1 and a healthy carriers of HTLV-1 without exception (Figures 9A,C).

In ATL-21C(−), ATL-16T(−), and ATL-35T(−) T-cell lines, the *tax* gene transcript was detected by RT-PCR (Figure 9C) and real-time PCR (data not shown). No significant mutation(s) was detected in the *tax* gene integrated in these tumor-producing T-cell lines (data not shown). As reported, the *tax* gene expression was downregulated in most of the ATL cells *in vivo* and the leukemic cell lines (Figures 9B,C), although Tax was expressed in ATL-48T(+) leukemic cell line (Figure 9B). ED-70423C(+)/(−) cell lines expressed an aberrant size *tax* gene transcript of 80 amino acids (Aa) instead of the wild type Tax protein of 354 Aa via nucleotide sequence analysis (Figure 9B). The *tax* gene in ED-70423C cell line is assumed functionally inactive; however, it is not clear why ED-70423C(+)/(−) cell lines expressing such an aberrant *tax* mRNA was established.

The *HBZ* gene was expressed at a significant level in both the leukemic and non-leukemic T-cell lines without exception, indicating that *HBZ* gene expression is also required for the growth and malignant transformation of HTLV-1-infected T cells (Figure 9).

The *p53* gene was expressed at a significant level in most of the non-leukemic T-cell lines, but it was downregulated in the majority of ATL cell lines (Figure 9). No significant mutation(s) associated with p53 dysfunction were detected in ATL-21C(+)/(−) and ATL-16T(−) T-cell lines (data not shown).

## Discussion

Newborns breastfed by HTLV-1-infected mothers often became infected with HTLV-1 and developed ATL after a long latency period of > 40 years. A very long latency period and a low incidence of ATL make the elucidation of ATL pathogenesis challenging.

Because hematopoiesis is regulated by cytokines, these are believed to be involved in some hematopoietic malignancies. Indeed, IL-6 has been reported to be a key growth factor for myeloma cells, and IL-6-dependent multiple myeloma cell lines have been established (Treon and Anderson, 1998). Since the growth and function of normal T cells are physiologically regulated by the IL-2/IL-2R system, IL-2Rα constitutively expressed on ATL cells was considered to implicate the involvement of IL-2/IL-2R in the growth of ATL cells.

We have reported the establishment of 32 HTLV-1-infected T-cell lines, eight IL-2-dependent cell lines with clonality similar to that of ATL cells in patients (leukemic), and 24 IL-2-dependent T-cell lines with clonality differing from the ATL cells (non-leukemic), from the PBMCs of 26 ATL patients (Table 1) (Maeda et al., 1985, 1987). IL-2-dependent T-cell lines infected with HTLV-1 were also established from a patient with HAM/TSP, a Sézary syndrome-like skin disease with HTLV-1-infection, and two healthy carriers of HTLV-1 (Supplementary Table S1). HTLV-1-infected T cells, if not all, in ATL patients and the people with HTLV-1-infection were able to proliferate continuously expressing IL-2Rα in the presence of IL-2, but not in the absence IL-2.

Adult T-cell leukemia cells in the majority of ATL patients appeared unresponsive to IL-2; however, eight IL-2-dependent ATL cell lines were established from five patients out of 26 ATL patients. The establishment of leukemic cell lines from a significant number of ATL patients (5/26:19%) as compared with other types of leukemia should be noted. Especially, the establishment of four ATL cell lines from an ATL patient has shown that the IL-2 responsive ATL cells have survived in the patient’s peripheral blood over 3 years of the clinical course.

HTLV-1-infected T cells were able to proliferate continuously *in vitro* in the presence of IL-2; however, IL-2 should be provided for T cells infected with HTLV-1 to proliferate continuously and survive *in vivo*. The IL-2 mRNA was expressed in the PBMCs isolated from four out of eight ATL patients and an HTLV-1 carrier, indicating that HTLV-1-infected T cells produced IL-2 for themselves (Figure 4A). Our results have been supported by two reports. The ATL cells isolated from an ATL patient proliferated in an autocrine fashion depending on the IL-2 that was produced by themselves (Arima et al., 1986). The HTLV-1-infected T cells isolated from HAM/TSP patients and HTLV-1 carriers, but not PBMCs in ATL patients, produced IL-2 and proliferated in an autocrine fashion (Tendler et al., 1990). Moreover, mRNA of IL-7 and IL-15 was expressed in the PBMCs of ATL patients, HTLV-1 carriers and healthy people. IL-2 and IL-15, which are physiological molecules to regulate the T-cell growth and function (Waldmann, 2006), are maintained in the human blood at a certain level (Kleiner et al., 2013), suggesting the involvement of these growth factors in the growth and survival of HTLV-1-infected T cells.

The leukemic cells in the majority of ATL patients express CD4, CD25, HLD-DR, and Foxp3 mRNA, a key molecule of Treg cells (Sakaguchi et al., 2006), indicating that ATL cells are mostly composed of Treg cells infected with HTLV-1 (Matsubara et al., 2005). The growth and function of Treg cells are strictly under the control of IL-2/IL-2R system (Sakaguchi et al., 2006). Consequently, IL-2/IL-2R is assumed to play a role in the survival and growth of IL-2-responsive ATL cells and HTLV-1-infected T cells *in vivo*. In some ATL cell lines, however, Foxp3 mRNA was not detected and ATL cells of approximately 18% ATL patients were comprised of CD4(−)CD8(+), CD4(+)CD8(+), or CD4(−)CD8(−) T cells (Kamihira et al., 1992). ATL-43T(+) was established from cells of an ATL patient with CD4(−)CD8(−)CD25(+) leukemic cells.

**FIGURE 10:**
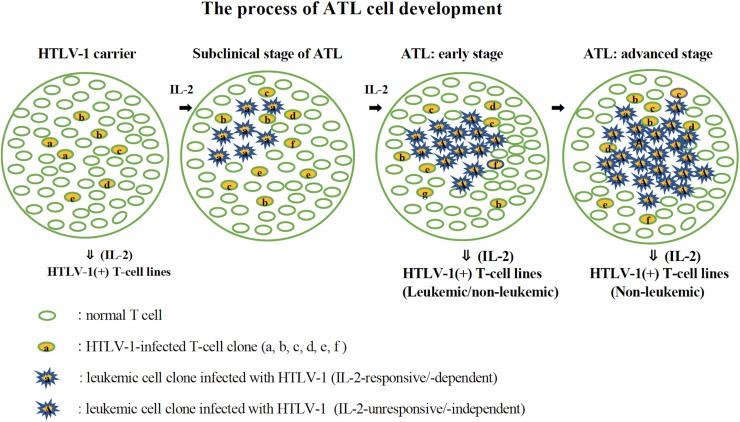
The process of ATL cell development. The possible process of the development of ATL cells from HTLV-1-infected T cells is shown. HTLV-1-infected T cells expressing IL-2 receptor proliferate in the presence of IL-2 (HTLV-1 carriers). A leukemic clone with multiple mutations responsible for malignant growth develops among them and expands its population depending on IL-2 (subclinical stage of ATL). An IL-2-independent leukemic clone develops among them and expands its population, leading to ATL (ATL: early and advanced stage). In the early stage of ATL, the leukemic cells are assumed to be mostly IL-2-independent/-unresponsive cells, but there are a few IL-2-dependent/-responsive leukemic cells. IL-2-dependent leukemic or non-leukemic cell line could be established from the PBMCs during this stage. In the advanced stage of ATL, leukemic cells are supposed to be IL-2-unresponsive, from which IL-2-dependent non-leukemic cell line, but not leukemic cell line, could be established.

Establishment of a significant number of IL-2-dependent leukemic T-cell lines from ATL patients suggests that a leukemic cell clone with critical genetic/epigenetic change(s) and/or gene expression responsible for the malignant transformation (Kataoka et al., 2015) originates among the T cells infected with HTLV-1 growing dependently on IL-2. The leukemic clone cells could survive and expand depending on IL-2 that is produced by themselves and/or produced as a physiological molecule to maintain normal T cells (Kleiner et al., 2013), and further evolve into ATL cells that proliferate without IL-2. The established IL-2-dependent leukemic cell lines might have been derived from the IL-2-responsive leukemic cell clones surviving among IL-2-unresponsive ATL cells. Such IL-2-dependent leukemic cells are assumed to be in an initiation stage of leukemia/cancer development. Based on these results, the process of ATL cell development is proposed in Figure 10. The involvement and importance of IL-2Rα in the development of ATL cells *in vivo* was confirmed by the clinical trials that anti-IL-2Rα (anti-Tac) antibody conjugated with radionuclide had a significant therapeutic effect on ATL patients (Waldmann, 2007).

It was previously reported that the transition from IL-2-dependent to IL-2-independent growth correlated with the acquisition of a constitutively activated Jak-STAT pathway (Migone et al., 1995). The constitutive binding of STAT was observed in the IL-2-dependent ATL-43T cells, indicating that these cells had already acquired an activated status of Jak-STAT pathway (Ahsan et al., unpublished results). Further investigation in other clones is required.

Interleukin-15 shares two of three subunits of IL-2R, IL-2Rβ, and γC, and was reported to promote the growth of IL-2-dependent T-cell line cells (Yamada et al., 1998). IL-15 was confirmed to be able to maintain the growth of IL-2-dependent ATL cell lines established by ourselves (Figure 5). IL-15 is produced ubiquitously by several cell types, such as dendritic cells and keratinocytes in the skin, where skin tumors or typical skin lesions infiltrated with ATL cells were commonly observed in ATL patients. Thus IL-15 may be involved in the proliferation of IL-2-responsive HTLV-1-infected T cells *in vivo*. IL-7 and IL-4 also promoted the growth of IL-2-dependent ATL-43T(+) cell line cells (Figure 5E). These results provided the supporting evidence for HTLV-1-infected T cells to be able to proliferate depending on IL-2/IL-15 and make malignant transformation to ATL cells *in vivo*.

The *tax* and *HBZ* genes were expressed in the *in vitro*-transformed HTLV-1-infected T cells, however, the *tax* gene was silenced or downregulated in the majority of ATL cells *in vivo* and the ATL cell lines (Figures 9B,C) (Takeda et al., 2004; Kataoka et al., 2015). Tax mRNA was expressed in the IL-2-dependent non-leukemic T-cell lines infected with HTLV-1 without exception (Figures 9A,C), indicating that the *tax* gene expression is required for IL-2-dependent proliferation of non-leukemic T-cell lines (Mahgoub et al., 2018), which is assumed to be the early stage of HTLV-1-infected T cells that finally make malignant transformation to ATL cells. However, the *tax* gene transcripts were not detectable or slightly expressed in two IL-2-dependent ATL cell lines, ATL-42T(+) and ED-40515(+), indicating that the *tax* gene expression may not be required for the IL-2-dependent growth of some ATL cell lines (Figure 9B).

Why was the *tax* gene expression downregulated in ATL cells but not in *in vitro*-transformed T cells? ATL cells and non-leukemic T cells infected with HTLV-1 express the Tax protein, which is the major target molecule of the cytotoxic T cells against HTLV-1-infected T cells (Jacobson et al., 1990; Kannagi et al., 1991). Cytotoxic T cells against HTLV-1-infected T cells could eliminate Tax(+) leukemic T cells, or downregulate the *tax* expression of ATL cells *in vivo*. ATL cells with downregulated Tax could survive and expand *in vivo*. Consequently, the *in vitro*-transformed T cells infected with HTLV-1 could maintain Tax expression because they are not exposed to immuno-surveillance *in vitro*. Although the Tax expression is controlled minimally in ATL cells *in vivo*, Tax protein is shown mandatory for the survival and leukemogenesis of HTLV-1-infected T cells (Mahgoub et al., 2018).

The *HBZ* gene was expressed in both leukemic and non-leukemic HTLV-1-infected T cells without exception, indicating that the HBZ protein plays an important role in the pathogenesis of ATL (Satou et al., 2011; Nakagawa et al., 2018).

Although the *p53* gene mutation was detected in a small population of ATL cells, *p53* was reported functionally suppressed by *tax* gene in ATL cells in less than half of ATL patients (Mahieux et al., 2000; Grassmann et al., 2005). The *tax* and *p53* genes were downregulated in most of the ATL leukemic cells, but not in the non-leukemic cells (Figures 9B,C).

In the process of malignant transformation, HTLV-1-infected T cells may be exposed to several complicated barriers such as immunological surveillance *in vivo*.

The downregulation or mutation of *p53* gene may be required for the malignant transformation of HTLV-1-infected T *in vivo* to overcome such barriers. However, the ATL-21C cells *in vitro* could have made malignant transformation by evading such barriers without p53 downregulation.

In the experiments to find out molecules differently expressed between IL-2-dependent and IL-2-independent T-cell lines infected HTLV-1, Thioredoxin-binding protein 2 (TBP-2/VDUP1/TXNIP) was found to be downregulated in the IL-2-independent but not in the IL-2-dependent HTLV-1-infected T-cell lines (Chen et al., 2011). Its significance remains to be elucidated.

Kataoka et al. (2015) performed an integrated molecular analysis of 426 ATL cases and found many gene mutations in ATL cells, which play a pivotal role in the malignant transformation of HTLV-1-infected T-cells. Accumulation of many gene mutations in HTLV-1-infected T-cells is thought to occur during the growth of HTLV-1-infected T cells, as shown in Figure 10. Moreover, they found many gene mutations on the T cell-related signaling, by which the role of IL-2/IL-2R in ATL pathogenesis is expected to be clarified.

The *in vitro* model proposed here for the pathogenesis of ATL could contribute in the better understanding of the molecular events involved in the development of ATL (Figure 10). These leukemic cell lines have been used to investigate the molecular mechanism and the therapeutic agents effective for the therapy of ATL (Zhang et al., 2015; Perera et al., 2017; Nakagawa et al., 2018). These tumorigenic leukemic T-cell lines could also be used to search the drugs for the treatment of ATL.

## Conclusion

Adult T-cell leukemia cells express IL-2Rα constitutively, which made it possible to establish eight leukemic and 24 non-leukemic T-cell lines growing dependently on IL-2 from the PBMCs of 26 ATL patients using IL-2. IL-2-dependent ATL cell lines evolved into IL-2-unresponsive ATL cells that proliferate without IL-2, resembling ATL cells *in vivo*. Three of the IL-2-dependent non-leukemic/normal T-cell line cells transformed into IL-2-independent tumor-producing cancer cells *in vitro*, along with three IL-2-independent leukemic T-cell lines.

HTLV-1-infected T cells of ATL patients appeared to produce IL-2 and support the proliferation and survival of the HTLV-1-infected T cells *in vivo*.

These results suggest that ATL cells develop among the HTLV-1-infected T cells growing dependently on IL-2, and that most of circulating ATL cells have progressed to become less responsive to IL-2, acquiring the ability to proliferate without IL-2.

## Materials and Methods

### ATL Patients and Blood Samples

Patients with ATL were diagnosed based on the clinical diagnosis criteria, hematological findings, and the presence of monoclonal T cells with integrated HTLV-1 provirus in peripheral blood (Shimoyama, 1991). All blood samples were obtained from the university hospitals, public and private hospitals in Japan between 1981 and 1999. Informed consents were obtained from patients and healthy HTLV-1 carriers of the facilities.

### Cell Cultures and IL-2

Mononuclear cells isolated from the peripheral blood of ATL patients or HTLV-1-infected donors using Ficoll-Hypaque solution (LSM solution; GE Healthcare Bio-Sciences Corp., Piscataway) were cultured at 1 × 10^5^ cells/ml in a 50 mm dish with RPMI1640 medium (Nacalai tesq, Kyoto) supplemented with 10% fetal bovine serum (FBS) and 100 μg/ml of kanamycin. Recombinant human IL-2 (Takeda Pharmaceutical Co., Osaka; Shionogi Pharmaceutical Co., Osaka; Suntory Co., Osaka; and PeproTec Inc., Rocky Hill) was added at 50 pM (7.5 ng/ml: 1 unit/ml). No other reagents except for IL-2 were used in our culture of HTLV-1-infected cell samples. Cells were cultured in a humidified atmosphere with 5% CO_2_ at 37°C. When cell density in culture was high enough, cultured cells were diluted by 1:2 to 1:8 with fresh culture medium in order not to dilute out the HTLV-1-infected T cells. Cells expressing IL-2Rα/CD25 and HLA-DR, the hallmarks of HTLV-1-infected T cells, were monitored using fluorescence tagged anti-IL-2Rα (anti-Tac) and anti-HLA-DR monoclonal antibodies (Maeda et al., 1985). Most of these T-cell lines established from ATL patients and non-ATL patients infected with HTLV-1 were deposited in the cell bank of RIKEN BioResource Center (BRC) of Japan.

### Southern Blot Analysis

High molecular weight DNA was digested with *Eco*RI or *Bam*HI, and later electrophoresed in a 0.6% agar gel, blotted to nitrocellulose filter paper and hybridized with ^32^P-labeled TCR β-chain (TCRβ) probe to examine the *TCRβ* gene rearrangement or with ^32^P-labeled HTLV-1 provirus probe to examine the provirus integration profile in genomic DNA as described (Maeda et al., 1985). Briefly, a 2.6 kb *Hin*dIII fragment of human *TCRβ* gene was used to detect *TCRβ* gene rearrangement and a 456 b *Sma*I*-Sac*I fragment of HTLV-1 provirus covering the long terminal repeat was used to detect the provirus integration. A leukemic cell line is defined as one showing the same southern blotting pattern of the TCRβ gene rearrangement and HTLV-1 provirus integration site as the patient’s leukemic cells. Non-leukemic cell line is defined as one that does not have the same southern blotting pattern as the patient’s leukemic cells. Thus, non-leukemic cell lines seem to have been derived from HTLV-I infected cells different from the patient’s leukemic cells.

### Inverse PCR

Clonality of the established T-cell lines was also examined by inverse PCR based on the analysis of the provirus integration site in the host genomic DNA (Etoh et al., 1997). The forward and reverse primers are shown in Supplementary Table S2.

### RT-PCR and Real-Time PCR

*Tax* expression in HTLV-1-infected T-cell lines was examined by RT-PCR and real-time PCR, as previously reported (Takeda et al., 2004). Real-time RT-PCR for *p53* gene was performed using the *p53* assay kit (TaqMan Gene Expression Assay kit, Hs01034249-m1, Applied BioSystems) according to the supplier’s instruction. Primer set used to detect the expression of genes are shown in Supplementary Table S2.

### Tax and p53 Genes Nucleotide Sequence Analysis

Coding sequences of *tax* and *p53* genes were analyzed using AB13100 DNA sequencer (Applied BioSystems).

### Tumorigenicity

Viable cultured cells were prepared at 1 × 10^6^–2 × 10^7^ cells/0.25 ml in Dulbecco’s PBS and inoculated subcutaneously into the back of BALB/c-nu/nu (Nude) and/or CB-17/IcrCrl-scid/scid (SCID) male mice. In some mice, cells were inoculated subcutaneously into their backs on both sides. Cells were inoculated into more than four sites or four mice to assay the tumorigenicity of each cell line as shown in Table 3. Mice were observed for 1–1.5 months after cell inoculation and tumor mass weight was measured. Male nude mice, 4–6-week-old, were obtained from the Institute of Laboratory Animals, Faculty of Medicine Kyoto University, and Charles River Laboratories Japan, Inc. Male SCID mice, 4–6-week-old, were obtained from Charles River Laboratories Japan, Inc. All animal experiments were performed in accordance with Kyoto University Animal Care and Use Committee guideline and were approved by the Committee.

### FACS Analysis

Cell surface phenotype was examined with mouse monoclonal antibodies against human cell surface antigen(s) obtained mostly from Nichirei Co., Tokyo. CD25 antibody was kindly provided by Dr. Uchiyama of Kyoto University Medical School. Fluorescein-isothiocyanate (FITC)-conjugated goat anti-mouse IgG was obtained from Cappel Co./ICN Pharmaceutical Inc. (Costa Mesa, United States). Viable cells were incubated with 20 μl monoclonal antibody for 30 min on ice, washed twice with Hanks’ buffer solution, and then incubated with 20 μl FITC-conjugated secondary antibody for 30 min on ice. Stained cells were examined by fluorescent microscopy and by fluorescence activated cell analyzer/sorter (FACS) machine (FACSCalibur or FACSCanto, BD Biosciences, Franklin Lakes, NJ, United States).

## Data Availability Statement

The raw data supporting the conclusions of this article will be made available by the authors, without undue reservation, to any qualified researcher.

## Ethics Statement

The studies involving human participants were reviewed and approved by the Kyoto University Graduate School and Faculty of Medicine, Ethics Committee. The patients/participants provided their written informed consent to participate in this study. The animal study was reviewed and approved by the Kyoto University Animal Care and Use Committee.

## Author Contributions

MiM designed the study. MiM, MaM, JY, and AS designed the experimental details. MiM and JT-S performed the experiments. MiM, KU, and MaM collected the clinical materials and data. MiM, MaM, JY, and HM discussed the experimental data. MiM, JT-S, PM, HM, and JY wrote the manuscript. All authors reviewed and approved the manuscript.

## Conflict of Interest

The authors declare that the research was conducted in the absence of any commercial or financial relationships that could be construed as a potential conflict of interest.

## Supplementary Material

FIGURE S1 Profiles of *TCRβ* chain gene rearrangement of three IL-2-independent ED-series leukemic cell lines derived from a patient. *TCRβ* chain gene rearrangement profile examined by Southern blot analysis indicates that these ED-series leukemic cell lines are derived from the ATL cells of the patient at different clinical stages. Lane 1: Human placenta (control), 2: primary PBMCs isolated from the ATL patient, 3: ED-40515(−) cell line, 4: ED-40810S(−), and 5: ED-41214C(−). DNA was digested with *EcoR1* and with *BamH1*. The reproducibility of the results was confirmed by performing the experiment more than twice.

FIGURE S2 Phenotype of ATL-21C cell line in the IL-2-dependent, IL-2-independent growth phases at 2 years, 3 years 8 months, and 8 years 9 months in culture, as analyzed by FACS. **(a)** ATL-21C(+). **(b)** ATL-21C(−)/2. **(c)** ATL-21C(−)/3.8. **(d)** ATL-21C(−)/8.9.

TABLE S1 HTLV-1-infected T-cell lines established from non-ATL patients and healthy people infected with HTLV-1.

TABLE S2 Primer set used for PCR.

## Abbreviations

ATL: adult T-cell leukemia
HAM/TSP: HTLV-1-associated myelopathy/tropical spastic paraparesis
HBZ: HTLV-1 bZIP factor
HTLV-1: human T-cell leukemia virus type 1
IL-2/IL-2R: interleukin-2/interleukin-2 receptor
IL-15: interleukin-15
PBMC: peripheral blood mononuclear cell
RT-PCR: reverse transcription polymerase chain reaction
SCID: severe combined immunodeficient
Tax: transactivator from the X-gene region of HTLV-1
TCR: T-cell receptor
Treg: regulatory T cell
WBC: white blood cell count.

## References

[B1] Arima N. Daitoku Y. Ohgaki S. Fukumori J. Tanaka H. Yamamoto Y. (1986). Autocrine growth of interleukin 2-producing leukemic cells in a patient with adult T cell leukemia. *Blood* 68 779–782. 10.1182/blood.v68.3.779.bloodjournal683779 2874849

[B2] Boxus M. Twizere J. C. Legros S. Dewulf J. F. Kettmann R. Willems L. (2008). The HTLV-1 Tax interactome. *Retrovirology* 5:76. 10.1186/1742-4690-5-76 18702816 PMC2533353

[B3] Chen Z. Lopez-Ramos D. A. Yoshihara E. Maeda Y. Masutani H. Sugie K. (2011). Thioredoxin-binding protein-2 (TBP-2/VDUP1/TXNIP) regulates T-cell sensitivity to glucocorticoid during HTLV-I-induced transformation. *Leukemia* 25 440–448. 10.1038/leu.2010.286 21151022 PMC3072512

[B4] Etoh K. Tamiya S. Yamaguchi K. Okayama A. Tsubouchi H. Ideta T. (1997). Persistent clonal proliferation of human T-lymphotropic virus type I-infected cells in vivo. *Cancer Res.* 57 4862–4867. 9354450

[B5] Gallo R. C. (2005). The discovery of the first human retrovirus: HTLV-1 and HTLV-2. *Retrovirology* 2:17. 15743526 10.1186/1742-4690-2-17 PMC555587

[B6] Gazdar A. F. Carney D. N. Bunn P. A. Russell E. K. Jaffe E. S. Schechter G. P. (1980). Mitogen requirements for the in vitro propagation of cutaneous T-cell lymphomas. *Blood* 55 409–417. 10.1182/blood.v55.3.409.bloodjournal553409 6244013

[B7] Gillet N. A. Malani N. Melamed A. Gormley N. Carter R. Bentley D. (2011). The host genomic environment of the provirus determines the abundance of HTLV-1-infected T-cell clones. *Blood* 117 3113–3122. 10.1182/blood-2010-10-312926 21228324 PMC3062313

[B8] Grassmann R. Aboud M. Jeang K. T. (2005). Molecular mechanisms of cellular transformation by HTLV-1 Tax. *Oncogene* 24 5976–5985. 10.1038/sj.onc.1208978 16155604

[B9] Grassmann R. Dengler C. Muller-Fleckenstein I. Fleckenstein B. McGuire K. Dokhelar M. C. (1989). Transformation to continuous growth of primary human T lymphocytes by human T-cell leukemia virus type I X-region genes transduced by a Herpesvirus saimiri vector. *Proc. Natl. Acad. Sci. U.S.A.* 86 3351–3355. 10.1073/pnas.86.9.3351 2541443 PMC287130

[B10] Grossman W. J. Kimata J. T. Wong F. H. Zutter M. Ley T. J. Ratner L. (1995). Development of leukemia in mice transgenic for the tax gene of human T-cell leukemia virus type I. *Proc. Natl.Acad. Sci. U.S.A.* 92 1057–1061. 10.1073/pnas.92.4.1057 7862633 PMC42636

[B11] Hasegawa H. Sawa H. Lewis M. J. Orba Y. Sheehy N. Yamamoto Y. (2006). Thymus-derived leukemia-lymphoma in mice transgenic for the Tax gene of human T-lymphotropic virus type I. *Nat. Med.* 12 466–472. 10.1038/nm1389 16550188

[B12] Hattori T. Uchiyama T. Toibana T. Takatsuki K. Uchino H. (1981). Surface phenotype of Japanese adult T-cell leukemia cells characterized by monoclonal antibodies. *Blood* 58 645–647. 10.1182/blood.v58.3.645.645 6455129

[B13] Hinuma Y. Nagata K. Hanaoka M. Nakai M. Matsumoto T. Kinoshita K. I. (1981). Adult T-cell leukemia: antigen in an ATL cell line and detection of antibodies to the antigen in human sera. *Proc. Natl Acad. Sci. U.S.A.* 78 6476–6480. 10.1073/pnas.78.10.6476 7031654 PMC349062

[B14] Hoshino H. Esumi H. Miwa M. Shimoyama M. Minato K. Tobinai K. (1983). Establishment and characterization of 10 cell lines derived from patients with adult T-cell leukemia. *Proc. Natl.Acad. Sci.U. S.A.* 80 6061–6065. 10.1073/pnas.80.19.6061 6193528 PMC534360

[B15] Iwanaga M. Watanabe T. Yamaguchi K. (2012). Adult T-cell leukemia: a review of epidemiological evidence. *Front. Microbiol.* 3:322. 10.3389/fmicb.2012.00322 22973265 PMC3437524

[B16] Jacobson S. Shida H. McFarlin D. E. Fauci A. S. Koenig S. (1990). Circulating CD8+ cytotoxic T lymphocytes specific for HTLV-I pX in patients with HTLV-I associated neurological disease. *Nature* 348 245–248. 10.1038/348245a0 2146511

[B17] Kamihira S. Sohda H. Atogami S. Toriya K. Yamada Y. Tsukazaki K. (1992). Phenotypic diversity and prognosis of adult T-cell leukemia. *Leukemia Res.* 16 435–441. 10.1016/0145-2126(92)90168-7 1625468

[B18] Kannagi M. Harada S. Maruyama I. Inoko H. Igarashi H. Kuwashima G. (1991). Predominant recognition of human T cell leukemia virus type I (HTLV-I) pX gene products by human CD8+ cytotoxic T cells directed against HTLV-I-infected cells. *Int. Immunol.* 3 761–767. 10.1093/intimm/3.8.761 1911545

[B19] Kataoka K. Nagata Y. Kitanaka A. Shiraishi Y. Shimamura T. Yasunaga J. (2015). Integrated molecular analysis of adult T cell leukemia/lymphoma. *Nat. Genet.* 47 1304–1315. 10.1038/ng.3415 26437031

[B20] Katoh T. Harada T. Morikawa S. Wakutani T. (1986). IL-2- and IL-2-R- independent proliferation of T-cell lines from adult T-cell leukemia/lymphoma patients. *Int. J. Cancer* 38 265–274. 10.1002/ijc.2910380218 2874115

[B21] Kleiner G. Marcuzzi A. Zanin V. Monasta L. Zauli G. (2013). Cytokine levels in the serum of healthy subjects. *Med. Inflamm.* 2013:434010. 10.1155/2013/434010 23533306 PMC3606775

[B22] Maeda M. Arima N. Daitoku Y. Kashihara M. Okamoto H. Uchiyama T. (1987). Evidence for the interleukin-2 dependent expansion of leukemic cells in adult T cell leukemia. *Blood* 70 1407–1411. 10.1182/blood.v70.5.1407.bloodjournal7051407 2889484

[B23] Maeda M. Shimizu A. Ikuta K. Okamoto H. Kashihara M. Uchiyama T. (1985). Origin of human T-lymphotrophic virus I-positive T cell lines in adult T cell leukemia. Analysis of T cell receptor gene rearrangement. *J. Exp.Med.* 162 2169–2174. 10.1084/jem.162.6.2169 2866223 PMC2187984

[B24] Mahgoub M. Yasunaga J. I. Iwami S. Nakaoka S. Koizumi Y. Shimura K. (2018). Sporadic on/off switching of HTLV-1 Tax expression is crucial to maintain the whole population of virus-induced leukemic cells. *Proc. Natl. Acad. Sci. U.S.A.* 115 E1269–E1278. 10.1073/pnas.1715724115 29358408 PMC5819419

[B25] Mahieux R. Pise-Masison C. A. Nicot C. Green P. Hall W. W. Brady J. N. (2000). Inactivation of p53 by HTLV type 1 and HTLV type 2 Tax trans-activators. *AIDS Res.Hum.Retrovi.* 16 1677–1681. 10.1089/08892220050193137 11080809

[B26] Matsubara Y. Hori T. Morita R. Sakaguchi S. Uchiyama T. (2005). Phenotypic and functional relationship between adult T-cell leukemia cells and regulatory T cells. *Leukemia* 19 482–483. 10.1038/sj.leu.2403628 15674359

[B27] Migone T. Lin J. Cereseto A. Mulloy J. O’Shea J. Franchini G. (1995). Constitutive activated Jak-STAT pathway in T-cells transformed with HTLV-I. *Science* 269 79–81. 10.1126/science.7604283 7604283

[B28] Miyoshi I. Kubonishi I. Sumida M. Hiraki S. Tsubota T. Kimura I. (1980). A novel T-cell line derived from adult T-cell leukemia. *Gann* 71 155–156. 6966589

[B29] Miyoshi I. Kubonishi I. Yoshimoto S. Akagi T. Ohtsuki Y. Shiraishi Y. (1981a). Type C virus particles in a cord T-cell line derived by co-cultivating normal human cord leukocytes and human leukemic T cells. *Nature* 294 770–771. 10.1038/294770a0 6275274

[B30] Miyoshi I. Kubonishi I. Yoshimoto S. Shiraishi Y. (1981b). A T-cell line derived from normal human cord leukocytes by co-culturing with human leukemic T-cells. *Gann* 72 978–981. 6281119

[B31] Nakagawa M. Shaffer A. L. III Ceribelli M. Zhang M. Wright G. Huang D. W. (2018). Targeting the HTLV-I-Regulated BATF3/IRF4 Transcriptional Network in Adult T Cell Leukemia/Lymphoma. *Cancer Cell* 34 e210. 10.1016/j.ccell.2018.06.014 30057145 PMC8078141

[B32] Okamoto T. Ohno Y. Tsugane S. Watanabe S. Shimoyama M. Tajima K. (1989). Multi-step carcinogenesis model for adult T-cell leukemia. *Jap. J. Cancer Res.* 80 191–195. 10.1111/j.1349-7006.1989.tb02289.x 2498254 PMC5917708

[B33] Perera L. P. Zhang M. Nakagawa M. Petrus M. N. Maeda M. Kadin M. E. (2017). Chimeric antigen receptor modified T cells that target chemokine receptor CCR4 as a therapeutic modality for T-cell malignancies. *Am. J. Hematol.* 92 892–901. 10.1002/ajh.24794 28543380 PMC5546946

[B34] Poiesz B. J. Ruscetti F. W. Gazdar A. F. Bunn P. A. Minna J. D. Gallo R. C. (1980). Detection and isolation of type C retrovirus particles from fresh and cultured lymphocytes of a patient with cutaneous T-cell lymphoma. *Proc. Natl.Acad. Sci. U.S.A.* 77 7415–7419. 10.1073/pnas.77.12.7415 6261256 PMC350514

[B35] Sakaguchi S. Ono M. Setoguchi R. Yagi H. Hori S. Fehervari Z. (2006). Foxp3+ CD25+ CD4+ natural regulatory T cells in dominant self-tolerance and autoimmune disease. *Immunol. Rev.* 212 8–27. 10.1111/j.0105-2896.2006.00427.x 16903903

[B36] Satou Y. Yasunaga J. Yoshida M. Matsuoka M. (2006). HTLV-I basic leucine zipper factor gene mRNA supports proliferation of adult T cell leukemia cells. *Proc. Natl. Acad. Sci. U.S.A.* 103 720–725. 10.1073/pnas.0507631103 16407133 PMC1334651

[B37] Satou Y. Yasunaga J. Zhao T. Yoshida M. Miyazato P. Takai K. (2011). HTLV-1 bZIP factor induces T-cell lymphoma and systemic inflammation in vivo. *PLoS Pathog.* 7:e1001274. 10.1371/journal.ppat.1001274 21347344 PMC3037353

[B38] Shimoyama M. (1991). Diagnostic criteria and classification of clinical subtypes of adult T-cell leukaemia-lymphoma. A report from the Lymphoma Study Group (1984-87). *Br. J. Haematol.* 79 428–437. 10.1111/j.1365-2141.1991.tb08051.x 1751370

[B39] Sugamura K. Fujii M. Kannagi M. Sakitani M. Takeuchi M. Hinuma Y. (1984). Cell surface phenotypes and expression of viral antigens of various human cell lines carrying human T-cell leukemia virus. *Int. J. Cancer* 34 221–228. 10.1002/ijc.2910340213 6088403

[B40] Takeda S. Maeda M. Morikawa S. Taniguchi Y. Yasunaga J. Nosaka K. (2004). Genetic and epigenetic inactivation of tax gene in adult T-cell leukemia cells. *Int. J. Cancer* 109 559–567. 10.1002/ijc.20007 14991578

[B41] Tendler C. L. Greenberg S. J. Blattner W. A. Manns A. Murphy E. Fleisher T. (1990). Transactivation of interleukin 2 and its receptor induces immune activation in human T-cell lymphotropic virus type I-associated myelopathy: pathogenic implications and a rationale for immunotherapy. *Proc. Natl. Acad. Sci. U.S.A.* 87 5218–5222. 10.1073/pnas.87.13.5218 2367534 PMC54293

[B42] Treon S. P. Anderson K. C. (1998). Interleukin-6 in multiple myeloma and related plasma cell dyscrasias. *Curr. Opin. Hematol.* 5 42–48. 9515202

[B43] Uchiyama T. Yodoi J. Sagawa K. Takatsuki K. Uchino H. (1977). Adult T-cell leukemia: clinical and hematologic features of 16 cases. *Blood* 50 481–492. 10.1182/blood.v50.3.481.bloodjournal503481 301762

[B44] Waldmann T. A. (2006). The biology of interleukin-2 and interleukin-15: implications for cancer therapy and vaccine design. *Nat. Rev. Immunol.* 6 595–601. 10.1038/nri1901 16868550

[B45] Waldmann T. A. (2007). Daclizumab (anti-Tac. Zenapax) in the treatment of leukemia/lymphoma. *Oncogene* 26 3699–3703. 10.1038/sj.onc.1210368 17530023

[B46] Yamada Y. Sugawara K. Hata T. Tsuruta K. Moriuchi R. Maeda T. (1998). Interleukin-15 (IL-15) can replace the IL-2 signal in IL-2-dependent adult T-cell leukemia (ATL) cell lines: expression of IL-15 receptor alpha on ATL cells. *Blood* 91 4265–4272. 10.1182/blood.v91.11.4265.411k06_4265_4272 9596675

[B47] Yodoi J. Takatsuki K. Masuda T. (1974). Letter: Two cases of T-cell chronic lymphocytic leukemia in Japan. *N.Eng. J. Med.* 290 572–573. 10.1056/nejm197403072901019 4544052

[B48] Yoshida M. Miyoshi I. Hinuma Y. (1982). Isolation and characterization of retrovirus from cell lines of human adult T-cell leukemia and its implication in the disease. *Proc. Natl. Acad. Sci. U.S.A.* 79 2031–2035. 10.1073/pnas.79.6.2031 6979048 PMC346116

[B49] Yoshida M. Seiki M. Yamaguchi K. Takatsuki K. (1984). Monoclonal integration of human T-cell leukemia provirus in all primary tumors of adult T-cell leukemia suggests causative role of human T-cell leukemia virus in the disease. *Proc. Natl. Acad. Sci. U.S.A.* 81 2534–2537. 10.1073/pnas.81.8.2534 6326131 PMC345097

[B50] Zhang M. Mathews Griner L. A. Ju W. Duveau D. Y. Guha R. Petrus M. N. (2015). Selective targeting of JAK/STAT signaling is potentiated by Bcl-xL blockade in IL-2-dependent adult T-cell leukemia. *Proc. Natl. Acad. Sci. U.S.A.* 112 12480–12485. 10.1073/pnas.1516208112 26396258 PMC4603455

