# Supplementary material for: IL-2/IL-2 Receptor Pathway Plays a Crucial Role in the Growth and Malignant Transformation of HTLV-1-Infected T Cells to Develop Adult T-Cell Leukemia
Source: Front Microbiol. 2020 Mar 6;11:356. doi: 10.3389/fmicb.2020.00356 (PMC7067701; doi:10.3389/fmicb.2020.00356)
Supplement: FIGURE S1 — Profiles of TCRβ chain gene rearrangement of three IL-2-independent ED-series leukemic cell lines derived from a patient. TCRβ chain gene rearrangement profile examined by Southern blot analysis indicates that these ED-series leukemic cell lines are derived from the ATL cells of the patient at different clinical stages. Lane 1: Human placenta (control), 2: primary PBMCs isolated from the ATL patient, 3: ED-40515(−) cell line, 4: ED-40810S(−), and 5: ED-41214C(−). DNA was digested with EcoR1 and with BamH1. The reproducibility of the results was confirmed by performing the experiment more than twice. [file Presentation_1.PPTX]

## Slide 1
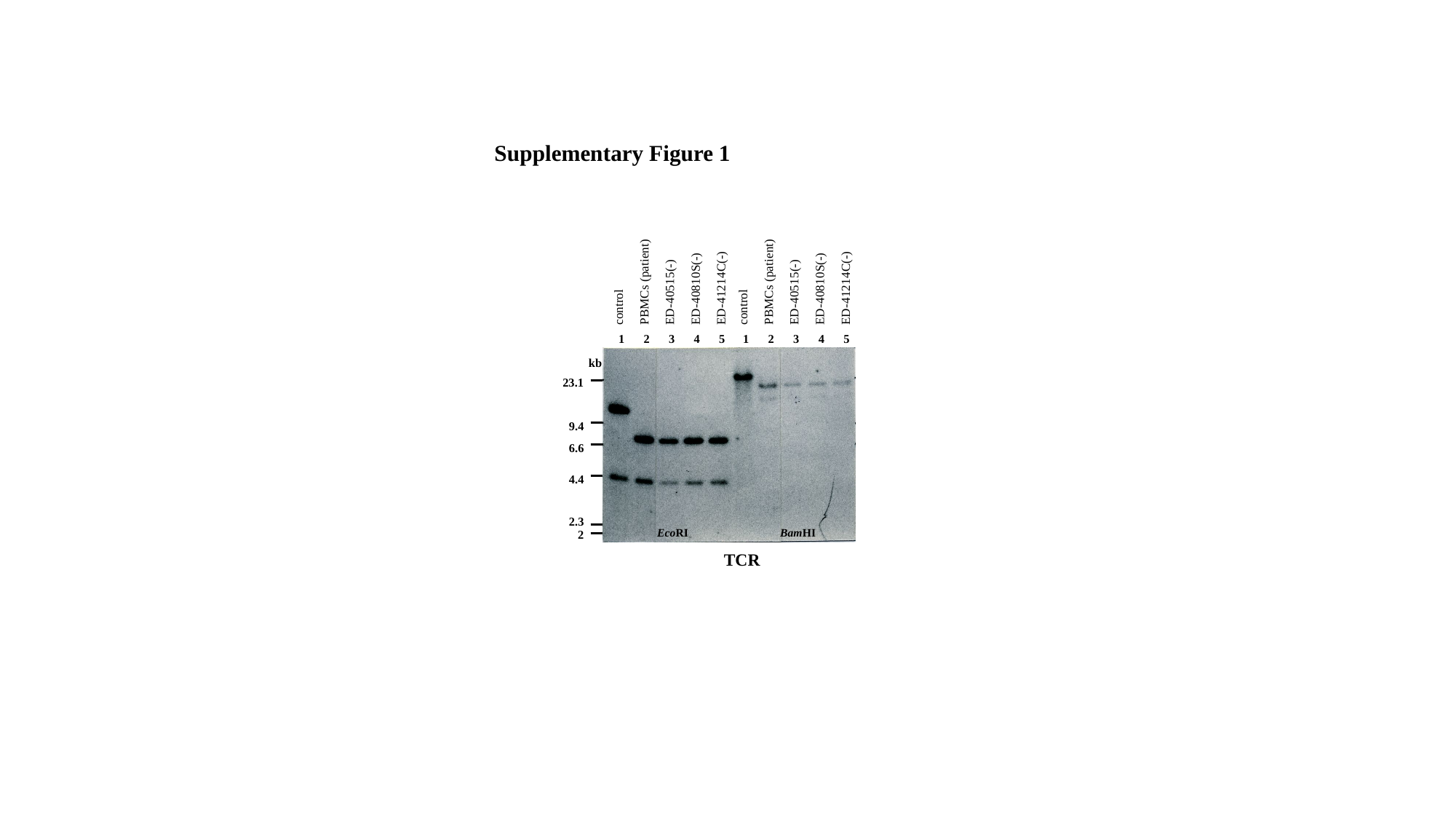

Supplementary Figure 1
PBMCs (patient)
PBMCs (patient)
ED-41214C(-)
ED-41214C(-)
ED-40810S(-)
ED-40810S(-)
ED-40515(-)
ED-40515(-)
control
control
1
2
3
4
5
1
2
3
4
5
kb
23.1
9.4
6.6
4.4
2.3
EcoRI
BamHI
2
TCR

## Slide 2
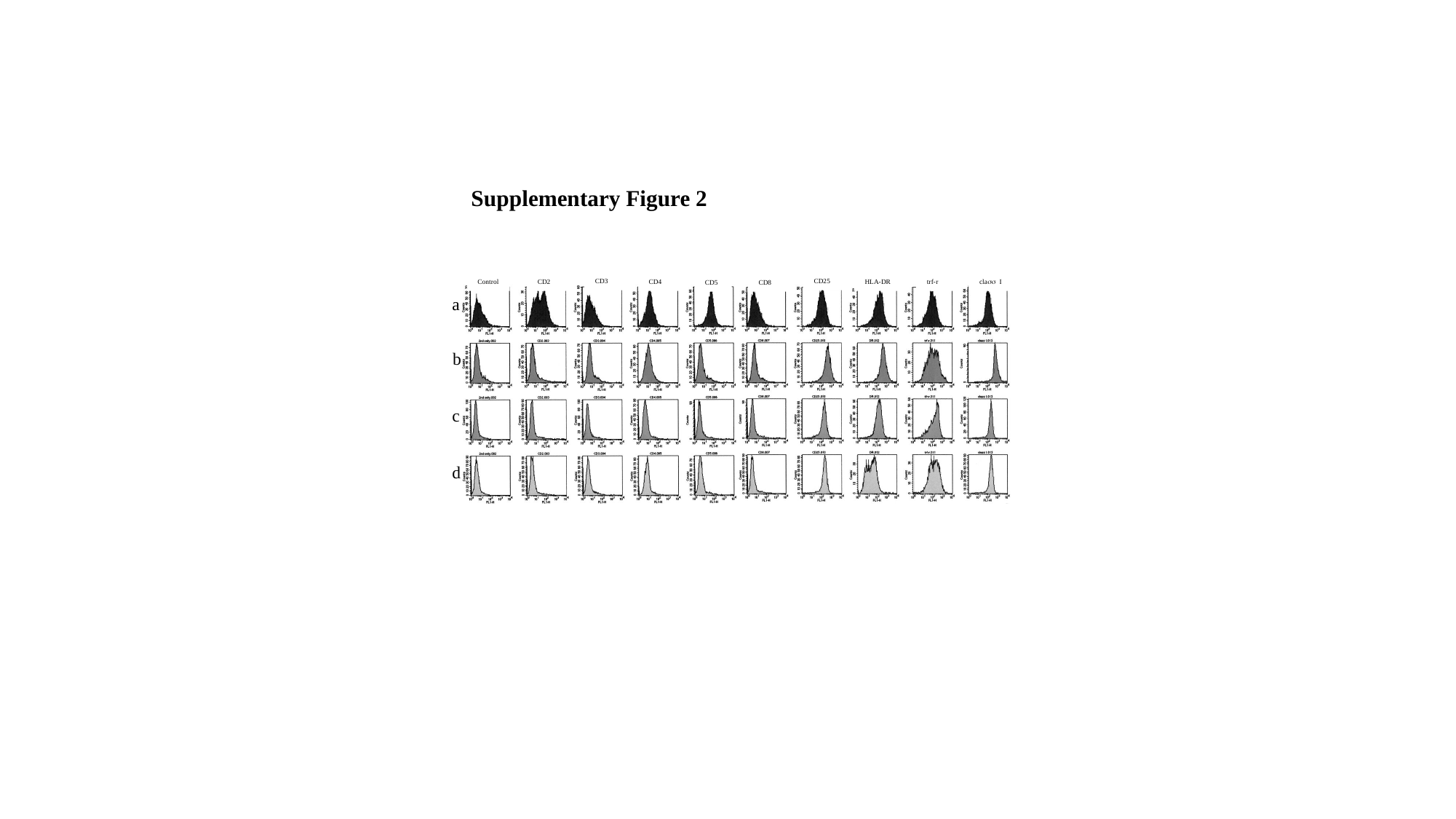

Supplementary Figure 2
CD3
CD25
Control
CD2
CD4
HLA-DR
trf-r
class I
CD5
CD8
a
b
c
d
