# Supplementary material for: IL-2/IL-2 Receptor Pathway Plays a Crucial Role in the Growth and Malignant Transformation of HTLV-1-Infected T Cells to Develop Adult T-Cell Leukemia
Source: Front Microbiol. 2020 Mar 6;11:356. doi: 10.3389/fmicb.2020.00356 (PMC7067701; doi:10.3389/fmicb.2020.00356)
Supplement: TABLE S1 — HTLV-1-infected T-cell lines established from non-ATL patients and healthy people infected with HTLV-1. [file Table_1.DOCX]

**Supplementary Table 1.**

**HTLV-1-infected T-cell lines established from non-ATL patients and healthy people infected with HTLV-1**

| Cell line name | Patient or Carrier No. | Source of the cells | Conversion to IL-2-independent | Phenotype CD4/CD8 |
| --- | --- | --- | --- | --- |
| HAM1C | #1 | HAM/TSP | - | CD4+ |
| Sez507 | #2 | Erythroderma | - | CD4+ |
| Sez627 | #2 | Erythroderma | + | CD4-/CD8- |
| SezM | #3 | Healthy carrier | - | CD4+ |
| SezM3 | #3 | Healthy carrier | + | CD4+ |
| CATL-3 | #4 | Healthy carrier | - | CD4+ |

HTLV-1(+) T-cell lines were established in the presence of IL-2 from the peripheral blood mononuclear cells of a patient with HAM/TSP (HAM1C), a patient with Sézary syndrome-like disease infected with HTLV-1 (Sez507 and Sez627) and two healthy HTLV-1 carriers (SezM, SezM3 and CATL-3). Conversion to IL-2-independency and the phenotype (CD4/CD8) of the cell lines are shown.
