# Supplementary material for: IL-2/IL-2 Receptor Pathway Plays a Crucial Role in the Growth and Malignant Transformation of HTLV-1-Infected T Cells to Develop Adult T-Cell Leukemia
Source: Front Microbiol. 2020 Mar 6;11:356. doi: 10.3389/fmicb.2020.00356 (PMC7067701; doi:10.3389/fmicb.2020.00356)
Supplement: TABLE S2 — Primer set used for PCR. [file Table_2.DOCX]

**Supplementary Table 2 Primer set used for PCR**

gene

*tax*

*TP53*

*HBZ*

*GAPDH*

Forward primer

5’-CCGGCGCTGCTCTCATCCCGGT-3’

5’-GTGAGCGCTTCGAGATGTTCC-3’

5’-AGGGCTGTTTCGATGCTTGC-3’

5’-ATGGGGAAGGTGAAGGTCGGAGTC-3’

Reverse primer

5’-GGCCGAACATAGTCCCCCAGAG-3’

5’-TTCAACAGTGAGGGACAGCTTC-3’

5’-CTGCCGATCACGATGCGTTT-3’

5’-CCATGCCAGTGAGCTTCCCGTTC-3’

**RT-PCR**

**Primer set to detect cytokine and its receptor**

Reverse primer

5’-GTCAGTGTTGAGATGATGCTTTGAC-3’

5’-CGAACACTTTGAATATTTCTCTCTCAT-3’

5’-TGCATTTCTCAAATGCCCTAATCCG-3’

5’-TCTTTACAGTCGCGCAACGGA-3’

5’-CCTCACATTCTTTGCATCCAG-3’

5’-TCTCCTGGGCGACCATTTAGC-3’

5’-AGTCCGCTTCCAAGAGTTTCC-3’

5’-ACTTAGGGCTACAGGACCCTG-3’

5’-GGTTAGTAAGATAGGATCCAT-3’

5’-TTCCATTTCAACGCTGGCCAG-3’

5’-AGACAGCACTGTGTTGGCGAT-3’

Forward primer

5’-ATGTACAGGATGCAACTCCTGTCTT-3’

5’-ATGGGTCTCACCTCCCAACTGCT-3’

5’-ATGTTCCATGTTTCTTTTAGGTATATCT-3’

5’-AAGATCCAGCTTCCAAGTGCC-3’

5’-TGTTTCAGTGCAGGGCTTCCT-3’

5’-TCAGTGCGTCCAGGGATAG-3’

5’-ACCCTGTGGATGTAATGGCGG-3’

5’-CGCCATGTTGAAGCCATCATT-3’

5’-AATGGAGACTTGGAAGATGCA-3’

5’-TGAACAAGGCCACGAATGTCG-3’

5’-CACTGGCATCGTGATGGACT-3’

*IL2*

*IL4*

*IL7*

*IL9*

*IL15*

*IL2Rα*

*IL2Rβ*

*IL2Rγ*

*IL7R*

*IL15Rα*

*β-actin*

gene

**I-PCR**

Restriction enzyme

*Pst*-I

*Hin*d-III

Forward primer

5'-CTCAATAAACTAGCAGGAGTCTATAAAAGCG-3'

5'-CTCAATAAACTAGCAGGAGTCTATAAAAGCG-3'

Reverse primer

5'-CAGTCGCCTTGTACACAGTCTCCAAACACG-3'

5'-AAATCAAAGTGGCGAGAAACTTACCCATGGTG-3'

TaqMan probe

5’FAM-AAGACCACCAACACCATGGCCCA-TAMRA3’

5’FAM-CTTTGGTATCGTGGAAGGACTCATGACC-TAMRA3’

Forward primer

5’-CCGCCGATCCCAAAGAA-3’

5’-ACCAACTGCTTAGCACCCCT-3’

Reverse primer

5’-CTCTGTCCAAACCCTGGGAA-3’

5’-GTCTTCTGGGTGGCAGTGAT-3’

gene

*tax*

*GAPDH*

*TP53*

Applied Biosystems TaqMan(R) Gene Expression Assays kit (Assay ID: Hs01034249_m1)

**Real time PCR**
